# New York Medical and Surgical Society

**Published:** 1883-08-20

**Authors:** 


### NEW YORK MEDICAL AND SURGICAL SOCIETY.

A stated meeting was held February 10, 1883, Dr. T. Gaillard Thomas, president, in the chair.

RENAL CALCULUS.—Dr. H. F. Walker related a case as follows: The patient was a man, about forty-eight years of age, who had suffered periodically for at least ten years with attacks of pain in the left side in the region of the kidney, extending down toward the bladder, and with other symptoms of renal calculus. No calculus, however, had been passed in the urine. The attacks were preceded for two or three days by premonitory symptoms. Various kinds of treatment had been resorted to without much benefit. The last remedy administered was the fluid extract of hydrangea, in doses of a teaspoonful three times daily. After this had been taken for about two or three months the patient suffered from a specially severe attack of renal colic, which lasted for three days, being longer than ordinary, and the pain extended farther down the line of the ureter than usual. There was also nausea and vomiting. The attack was followed by the passage of a calculus about one-third the size of a Java coffee-bean, and the patient had since experienced complete relief. In reply to a question by Dr. Post, Dr. Walker said that hydrangea was moderately diuretic. He was led to use it in this case by an article in the Medical Record, in which the writer stated that it was almost a specific for renal calculi.

The President remarked that he had at present two cases of supposed renal calculus under observation in which he intended to administer hydrangea as a possible means of diagnosis. The first patient, the wife of a physician, had been seen by a specialist in genito-urinary diseases, who had sent her to him on account of a tumor which was perceptible on palpation over the abdominal walls. She had been taken very suddenly with violent pain in the back, which extended down the course of the ureter and the crural nerve. Soon afterward the urine became bloody, and contained a large amount of pus and epithelium from the pelvis of the kidney and the ureter. There were no tube casts. At the time of the occurrence of this sudden attack of pain the patient, or her husband, upon passing the hand over the abdomen, discovered a tumor. The tumor was quite distinct when Dr. Thomas saw the patient, but it was only perceptible in the sitting posture, a fact which he had observed in certain cases of displaced kidney. The presence of blood in the urine in considerable quantity suggested cancer of the kidney, but, as the disease had lasted for two years, and the patient was healthy-looking and growing stouter as the disease advanced, he thought malignant disease could be excluded. The affection in the second case had also lasted a long time, and gave a similar history.

Dr. A. C. Post remarked that a stone which had remained in the pelvis of the kidney for some time was usually too large to pass through the ureter.

The President remarked that it was astonishing how long a calculus might remain in the pelvis of the kidney for an indefinite period without giving rise to symptoms. Recently a physician, who died apparently with no other symptoms than gradual wasting away, was found at the autopsy to have a calculus in the pelvis of the kidney as long as the thumb, twice as large, and of the shape of an elephant.

Dr. Abram Dubois remarked that a man once brought to his office a handful of stones, some as large as the end of his finger, and stated that he had often passed them in his urine, and had not suffered in the least.

Dr. A. B. Ball reported further on the case of blood extravasation in the calf of the leg, narrated at a recent meeting of the society. He and Dr. Sands, who saw the case in consultation, had told the patient that the tumor would probably disappear within two weeks, and she would entirely recover. It had now been six weeks, and the patient had just got out of bed for the first time. The blood clot was probably more deeply situated than had been supposed, and, after having become hardened, was slowly undergoing absorption.

TORTICOLLIS.—Dr. Post stated that several years ago he reported a case ot torticollis in which he divided the sterno-cleidomastoid muscle by an open incision, division of the main bands of the muscle being followed by scarcely any relief whatever; the knife was then introduced more deeply, and the last layer of the muscle cut, with the effect of complete and permanent relief. He recently had a similar case in a girl who had suffered from torticollis in a marked degree from early infancy.

The President then read a paper on Intra-uterine Injections in the Treatment of Puerperal Septicæmia.

COMPLETE DESTRUCTION OF THE KIDNEY BY CANCEROUS DISEASE; REMOVAL.—The other case to which the President would call attention was that of a patient residing in Connectictu, who gave the following history: She was a married woman, fifty years of age, the mother of six or seven children. She stated that he had attended her twenty-five years ago with Dr. Metcalfe, and that seven years ago she had called at his office on account of a tumor situated on one side of the pelvis, and that he examined it and told her it was a fibrous tumor of the uterus. She had watched the tumor during these seven years, and it gave her no trouble until three months ago. It then began to increase in size, and continued to grow until it filled one-half of the abdominal cavity. The patient emaciated and lost strength, and had not been able to leave her bed during the last three months. Great doubt had been entertained by the physicians who had seen her with regard to the character of the tumor. An aspirator-needle had been passed, but no fluid was withdrawn. Dr. Thomas found, upon examination, that the tumor was of large size, and he was unable to determine whether it was ovarian or whether it was uterine. If it were uterine, it was probably the original fibroid tumor which, according to the patient's statement, he had diagnosticated some years previously, and which had since taken on the character of a sarcoma. As the patient would certainly die within a short time if an operation were not performed, he proceeded to remove the tumor eleven days ago. The moment the abdominal cavity was opened he recognized the fact that the tumor could not be ovarian, for the intestines lay in front of it. The uterus was also found to be entirely disconnected with the tumor. The tumor was evidently deeply situated behind the intestines, and fastened by adhesions, but he was unable, after repeated trials, turning the patient from side to side, to remove the intestines from its surface. The gentlemen present were consulted with regard to the propriety of closing the abdominal wound and abandoning the operation, but the general feeling was that if this were done the patient was doomed to certain death, and, although it was highly probable that death would take place after the removal of the tumor, it was thought better to give the patient the chances of the operation. He then cut through the mesocolon with the scissors, passed the hand down upon the tumor, gradually swept it upward, came upon the pedicle, which he ligated, and removed the tumor as quickly as possible, the condition of the patient now being very unfavorable. At that time he felt satisfied that the tumor was the kidney. The condition of the patient did not allow of time to search for the other kidney. The cavity was cleaned out, the opening closed, a large drainage-tube inserted, and the patient put to bed.

The specimen had been examined microscopically by Dr. Welch, who reported that the mass was composed of cancerous material, but he was unable to determine to what organ it belonged. He could only make out the renal capsule, which was also involved in the disease. Dr. Thomas felt certain, however, that the mass, which weighed six pounds, involved the kidney.

The stitches were removed on the ninth day. The patient did very well for forty-eight hours. She then became noisy, delirious, and at the end of fifty-six hours she was quite delirious, behaving very much like a person suffering from a low grade of puerperal mania. The pulse was between 130 and 135 before the operation; it had now fallen below 120. The urine had been secreted in amount from 24 to 26 ounces every day. There had been no tendency to suppuration, and no peritonitis, but there had evidently been some septicæmic action, the temperature having kept up to 102 and 103, and to-day was 101-6. It had at no time gone above 103. The patient would probably recover from the operation.

Discussion on Dr. Thomas's paper being in order, Dr. J. W. McLane said that his experience with washing out the uterus had been that, in those cases in which the absorption of the septic material had evidently taken place from within the uterus, the temperature could be reduced by injections into the cavity. But in some cases, undoubtedly septicæmic in character, the rapidity of the absorption was very great; a general peritonitis was set up, which would seem, at the post-mortem examination, to have been the only affection from which the patient had suffered. The question arose in his mind whether washing out the uterus under such circumstances would save the life of the patient.

The President thought that such cases would probably terminate fatally, whether the uterus were washed out or not; that it was then too late for treatment. His experience had been that the sooner the injections were begun in a case of puerperal fever the more successful would be the result.

Dr. G. G. Wheelock remarked that he had seen metro-peritonitis treated by washing out the uterine cavity with the result of not reducing the temperature in the least. The diagnosis had been confirmed by a post-mortem examination.

Dr. Walker remarked that he had made use of uterine injections a great deal, both in private and hospital practice, and had produced much benefit with them, especially in cases of threatening septicæmia.

The President remarked that Dr. Jones published a case several years ago, in which the injections were begun after the temperature had reached 107° and 108°, and the patient recovered.

Dr. Walker remarked that he had published a case in which the temperature rose to 108°, and injections were continued for six weeks before the patient fully recovered. He did not think we should always expect to find an external lesion which would account for the absorption of septic material; he believed that it took place more frequently from the inner surface.

The Piesideut referred to a case to which Dr. J. B. Hunter was called in consultation in a neighboring city about eleven days ago. The patient had been delivered five weeks previously, and was suflering from septicæmia with a temperature of 103-5°, the pulse 120. Her family physician, who was a competent man, and several others, had given up all hopes of saving the patient's life. When Dr. Hunter saw her he proposed to wash out the uterus, but was met with the most stubborn opposition. Obtaining the husband's consent, however, he proceeded to make the injections, and in less than twelve hours the temperature and the pulse fell, and the patient was greatly relieved. The treatment was still being continued. In reply to a question by Dr. McBurney, the

President said the strength of the solution which he used at the present time was 2½ per cent.

Dr. Wheelock remarked that at the Nursery and Child's Hospital, when the temperature rose to 103°, and there was a discharge of foul odor, uterine injections were used, and always with the most favorable results.

Dr. J. G. Curtis asked whether the cause of the entrance of air into the circulation in the cases which had been reported could, in every case, be traced directly to the introduction of the catheter into a uterine sinus.—*N. Y. Med. Record.*

